# Editorial: Advances in the potential treatments of gastrointestinal and liver diseases: addressing the public health burden, volume II

**DOI:** 10.3389/fphar.2026.1866943

**Published:** 2026-05-12

**Authors:** Adina Turcu-Stiolica, Maria Dimitrova, Mariana Jinga

**Affiliations:** 1 Department of Pharmacoeconomics, University of Medicine and Pharmacy of Craiova, Craiova, Romania; 2 Department of Organization and Economy of Pharmacy, Medical University of Sofia, Sofia, Bulgaria; 3 Department of Gastroenterology, Carol Davila University of Medicine and Pharmacy, Bucharest, Romania

**Keywords:** biomarker, gastrointesinal disorders, innovation, liver disease, polypharmacy

Gastrointestinal and liver diseases continue to pose a significant burden worldwide, both on societies and healthcare systems (Mar et al., 2018). With recent advances in studying disease pathogenesis and developing novel therapies with improved screening strategies for early detection, healthcare providers and policymakers can better address this major public health challenge (Goebel et al., 2015; Banales et al., 2026). The multifactorial nature of these disorders—spanning metabolic dysfunction, malignancy, inflammatory conditions, and complications of surgical interventions—demands integrative therapeutic strategies that bridge mechanistic discovery, clinical innovation, and health system optimization (Ionele et al., 2022). This Research Topic presents eleven contributions that advance our understanding of emerging therapeutic approaches, predictive models, and translational pathways in this rapidly evolving field. As a whole, they reflect the diversity of scientific inquiry needed to reduce the global disease burden.

A central theme emerging from this volume is the growing emphasis on targeting the mechanisms of liver injury pathways, particularly those involving oxidative stress, inflammation, and regulated cell death. Lei et al. provide a comprehensive bibliometric analysis revealing how research on hepatic ischemia–reperfusion injury (HIRI) has evolved over the decades, with oxidative stress consistently identified as a pivotal therapeutic target. Notably, the field is transitioning from descriptive mechanistic studies to intervention-oriented research, including nanotechnology-based approaches and epigenetic modulation. This shift underscores a broader trend across hepatology: the translation of molecular insights into actionable therapies. Complementing this mechanistic perspective, Chen et al. demonstrate that hydrogen gas administration attenuates steatosis, fibrosis, and inflammation in metabolic dysfunction–associated steatotic liver disease (MASLD) by inhibiting both canonical and non-canonical pyroptosis pathways. By linking redox homeostasis to inflammasome activation and gasdermin-mediated cell death, this study elegantly illustrates how targeting upstream oxidative stress yields downstream benefits across multiple pathogenic axes simultaneously.

Another important dimension explored in this Research Topic is liver regeneration and repair. Shen et al. investigate recombinant human thrombopoietin (rhTPO) as a novel platelet-driven regulator that accelerates hepatic regeneration in acute liver failure (ALF). Their preclinical studies demonstrate improved survival, enhanced hepatocyte proliferation via activation of MAPK cell proliferation signaling pathways, and reduced inflammatory gene expression. These findings not only expand the functional repertoire of platelets beyond their traditional hemostatic roles but also suggest that modulation of endogenous regenerative pathways could offer viable alternatives to liver transplantation, which remains severely limited by donor availability and substantial cost. Such approaches could potentially transform the management of acute hepatic decompensation in resource-limited settings.

Therapeutic innovation in gastrointestinal disorders is exemplified through several important contributions. Lu et al. characterize linaprazan glurate (X842), a novel potassium-competitive acid blocker (P-CAB), which demonstrates a rapid onset of action and prolonged inhibition of gastric acid secretion. By overcoming key limitations of proton pump inhibitors—such as delayed action and chemical instability—P-CABs represent an important pharmacological advancement that may redefine the management of acid-related diseases, including gastroesophageal reflux disease and peptic ulcer disease. This work exemplifies how incremental pharmacological refinement, informed by mechanistic understanding, can yield clinically meaningful improvements in therapeutic efficacy and patient outcomes. In the context of inflammatory bowel disease, Yu et al. present a real-world study evaluating rituximab in PR3-ANCA–positive ulcerative colitis patients—a biomarker-defined subgroup with limited responsiveness to conventional biologics—demonstrating superior clinical and endoscopic outcomes compared with infliximab. This contribution underscores the importance of stratified therapies in complex inflammatory conditions, where disease heterogeneity often limits treatment efficacy.

Cancer-related gastrointestinal diseases are another major focus of this Research Topic, spanning prevention, treatment, and healthcare economics. Shen et al. provide a comprehensive meta-analysis demonstrating that metformin significantly reduces the risk of colorectal neoplasms, with a dose-response relationship favoring low-dose, long-term therapy. This finding reinforces the substantial potential of repurposing widely used metabolic drugs for cancer chemoprevention, particularly in high-risk populations such as patients with diabetes mellitus. Conversely, Luo et al. assess the cost-effectiveness of ramucirumab plus paclitaxel in treating advanced gastric and gastroesophageal junction cancers, revealing that despite demonstrated clinical benefits, the regimen faces significant affordability barriers under current pricing structures in both China and the United States. This critical analysis highlights that aligning clinical efficacy with economic sustainability is essential for ensuring equitable access to innovative therapies worldwide.

Patient safety and optimization of clinical care are further advanced through sophisticated predictive modeling approaches (Ungureanu et al., 2023). Pei et al. develop a comprehensive risk prediction model for severe potential drug–drug interactions in colorectal cancer patients, identifying polypharmacy, hypoalbuminemia, and treatment complexity as key modifiable risk factors. Similarly, Sui et al. construct an inflammatory cytokine–based nomogram to predict prolonged postoperative ileus following radical gastrectomy, enabling clinicians to identify high-risk patients preoperatively. These studies exemplify the growing role of data-driven tools in personalized medicine, enabling early identification of high-risk patients and facilitating targeted preventive interventions to improve clinical outcomes. Beyond individual patient care, Polignano et al. analyze the distribution of gastrointestinal oncology clinical trials in Italy, revealing significant regional disparities directly linked to economic resources and healthcare infrastructure capacity (Pana et al., 2023). This important epidemiological analysis underscores the critical need for policy-level interventions to ensure equitable geographic access to innovative therapies.

Taken together, the contributions in this volume illustrate a truly multidimensional approach to addressing the substantial public health burden of gastrointestinal and liver diseases. Several cross-cutting themes emerge across these diverse contributions: (1) the integration of molecular mechanisms with therapeutic development is accelerating the translation of basic science discoveries into clinical applications; (2) precision medicine approaches—such as biomarker-guided therapies and sophisticated predictive modeling—are increasingly central to optimizing patient outcomes; and (3) critical considerations of cost-effectiveness and healthcare equity are essential to ensuring that advances in treatment translate into real-world population benefits. Looking forward, important challenges include validation of preclinical findings in diverse human populations, integration of multi-omics data into clinical decision-making algorithms, and the development of scalable healthcare delivery models. By bridging mechanistic insights, clinical innovation, and systemic considerations, this Research Topic provides a comprehensive roadmap for future research and underscores the importance of a holistic, multidisciplinary approach to reducing the global burden of gastrointestinal and liver diseases.
